# Bringing radiomics into a multi-omics framework for a comprehensive genotype–phenotype characterization of oncological diseases

**DOI:** 10.1186/s12967-019-2073-2

**Published:** 2019-10-07

**Authors:** Mario Zanfardino, Monica Franzese, Katia Pane, Carlo Cavaliere, Serena Monti, Giuseppina Esposito, Marco Salvatore, Marco Aiello

**Affiliations:** 1 IRCCS SDN, Naples, Italy; 2 Bio Check Up S.r.l., 80121 Naples, Italy

**Keywords:** Radiogenomics, MultiAssayExperiment, Radiomics, Cancer, TCGA, TCIA

## Abstract

Genomic and radiomic data integration, namely radiogenomics, can provide meaningful knowledge in cancer diagnosis, prognosis and treatment. Despite several data structures based on multi-layer architecture proposed to combine multi-omic biological information, none of these has been designed and assessed to include radiomic data as well. To meet this need, we propose to use the MultiAssayExperiment (MAE), an R package that provides data structures and methods for manipulating and integrating multi-assay experiments, as a suitable tool to manage radiogenomic experiment data. To this aim, we first examine the role of radiogenomics in cancer phenotype definition, then the current state of radiogenomics data integration in public repository and, finally, challenges and limitations of including radiomics in MAE, designing an extended framework and showing its application on a case study from the TCGA-TCIA archives. Radiomic and genomic data from 91 patients have been successfully integrated in a single MAE object, demonstrating the suitability of the MAE data structure as container of radiogenomic data.

## Background

Diseases are governed by complex biological mechanisms requiring different levels of analyses for a comprehensive interpretation of the underlying pathology. Today, the progress in genomics, transcriptomics, epigenomics and their combination, enables the incorporation of different biological layers of information to predict phenotypic conditions (tumor/normal, early/late stage, survival, etc.). Multi-omics data integration is, therefore, one of the major challenges in the era of precision medicine, particulary in oncology. With the huge increase in genomic data production, the need for specific models and methods for storing and analyzing those data has arisen; an example is MultiAssayExperiment (MAE) [1]. MAE handles multiple and heterogeneous data types for a set of samples of multi-assay genomic experiments (transcript counts, DNA variants or methylation status of genes or regions, etc.). However, these structures consider only data produced by molecular biology experiments and neglect the impact of other ‘omics which also deserve consideration. The progress that has been made in medical imaging techniques and the development of high-throughput algorithms to extract quantitative features from medical images has led to the development of radiomics. In clinical research, radiomics is becoming a meaningful tool and might be considered as an additional and complementary source of ‘omic information, not achievable in a multi-omics biological environment. In this scenario, the growing impact of non-invasive imaging techniques for disease definition, in parallel with the evolution of next-generation sequencing (NGS) tools, provides powerful methods for investigating the phenotype through the combination of imaging characteristics (radiomic features) into a multi-omics biological framework. Indeed, in recent years, correlation of radiomic features with genomic features, rise to a new field of study defined “radiogenomics” [2]. The increasing scale and availability of a high volume of health data requires new and efficient strategies for data management, data linkage and data integration. These types of datasets are defined “multimodal” [3] since multimodal signals are managed together. In this context, there are many challenges to overcome: identifying relationships between data from different modalities, joining multimodal information to execute prediction, learning information to help understand limited data of another modality and, crucial in our case, representing, integrating and summarizing multimodal data. Thus, in order to optimize data management and analysis, it is necessary to reshape the existing information systems into innovative multi-layer data systems by combining statistical and computational methods. So far, no tools integrating genomic and radiomic data have been designed; therefore, consolidating single-omic datasets from different domains in a meaningful manner is an ambitious undertaking. Here, we investigated the role of the MAE structure as a possible bridge for integrating radiomics into a multi-omics framework. To this end, we evaluated the potential of MAE as a structure for storing and managing both imaging and biological ‘omic data derived from different type of experiments, while keeping the coordinated representation of data and ensuring consistency between a single assay and clinical patient data during data subsetting and analysis intact. The extended multi-omics framework proposed here allows researchers to simplify the management of radiogenomic data. In this article, (i) we will first introduce the state of the art of both radiomics and biological ‘omics in the field of cancer research; (ii) we will then summarize the role of radiogenomics in cancer phenotype definition; (iii) we will discuss the current state of radiogenomic public repositories, their limits, challenges and limitations of including radiomics in a multi-omics framework; (iv) finally, we will demonstrate the feasibility of our approach with a case study using The Cancer Genome Atlas (TCGA), for biological data, and The Cancer Imaging Archive (TCIA), for public medical images.

## Radiomics and biological ‘omics in the field of cancer research: state of the art

### Radiomics framework

**Fig. 1:**
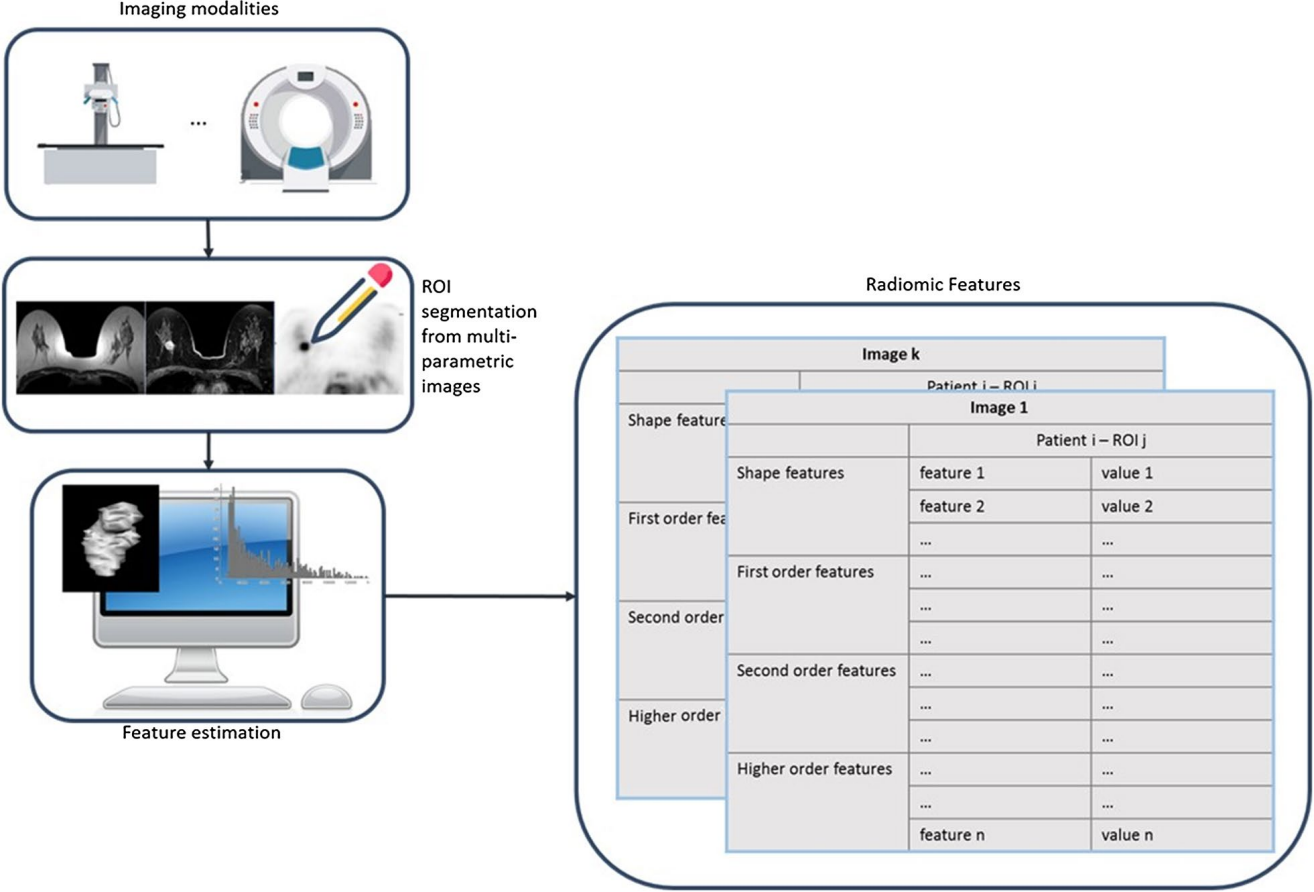
Radiomics workflow. Radiomics features can be calculated from one or more imaging modalities, e.g. computed tomography (CT), magnetic resonance (MR), positron emission tomography (PET), for each time point acquired. Then, regions of interest (ROIs) are segmented from the acquired multi-parametric images, e.g. T2 weighted MR image, Contrast Enhanced T1 weighted MR image, FDG PET image, as shown from left to right in the figure in a case of breast lesion. Finally, the radiomic features are estimated, providing hundreds of features that can be categorized as shape, first order, second order and higher order features, for each segmented ROI, for each patient in the study and for each acquired image

Radiomics arises from the increasing interest in the development of non-invasive diagnostic tools for disease characterization and monitoring, especially in cancer research [4, 5]. Diagnostic images are able to provide information on the entire tumor volume, reducing inaccuracy due to sampling errors in histopathological analyses. In this scenario, radiomics, i.e. the extraction of a large number of quantitative features from medical images [6], has proved to be a key way to study the cancer imaging phenotypes, reflecting underlying gene expression patterns [7, 8] and revealing heterogeneous tumor metabolism and anatomy [9, 10]. This high-throughput feature extraction is typically preparatory to a data mining process [11] in order to associate or predict different clinical outcomes [12], giving important prognostic information about the disease. Radiomics has the potential to extensively characterize the intratumoral heterogeneity, and it has shown promise in predicting treatment response and outcome, differentiating benign and malignant tumors and assessing the relationship with genetics in many cancer types [13–19]. The radiomic approach can be applied to any imaging modality, even on more modalities acquired at the same time point, providing multi-parametric features. Once the images are collected, the radiomic approach involves two main steps: the segmentation of Region Of Interest (ROI) and the estimation of descriptive features. ROI segmentation consists of the identification of target regions of prognostic value, which can be performed according to different strategies. After ROI segmentation, an automated process extracts quantitative features (descriptors) from each ROI. The descriptors are designed to provide information, related to the tumor phenotype and the microenvironment. Radiomic features can be divided into four groups: shape-based (geometric characteristics), first-order and second-order statistics features (texture characteristics) and higher-order features (impose filter grids on an image to extract repetitive or non-repetitive patterns to compute first- or second-order statistic features from). As a result, up to hundreds of features are obtained from a single image (Fig. 1). Finally, the extracted features, together with clinical or pathologic outcomes, are fed into machine-learning procedures to construct classification, predictive, or prognostic models [20].

### Biological multi-omics integration tools

In the past several years, various methods, data structures and tools, related to multi-omics data integration have been developed. For an exhaustive review of multi-omics data integration methods and a list of packages using these methods see Huang et al. [21]. In order to ensure structured relations between different layers of biological data, data containers are a necessary requirement. Some existing data structures for multi-omic assays have been utilized to meet this demand. Two of the most recent are MultiDataSet [22] and MultiAssayExperiment (MAE) [1] (R/Bioconductor packages). These packages manage several sets of biological experiments and facilitate the coordination of different types of operations, such as data visualization, data manipulation, subsetting, data integration and reshaping. Moreover, these data containers enable subsetting of data by different items, such as clinical or pathologic variables, genes, genomic ranges and assays. Additionally, data warehouses that enable users to dynamically interrogate clinico-pathologic data in a multidimensional manner are developed in this context. One such example is the Data Warehouse for Translational Research (DW4TR) [23].

We have chosen to test MAE as radiogenomic data container because of the extensive documentation, very frequent updating, integration of several R and Bioconductor data classes, ample set of data manipulation methods and a simplified graphical interface including many R/Bioconductor packages. Moreover, many of the available datasets, which include both radiomic and genomic data, are provided by TCGA and TCIA databases and the whole genomic part is already available as an MAE object. The structure of the MAE object makes possible coordinated operations through three main functions: *i)* reporting the property of sample units, such as clinical, pathological, and biospecimen data; *ii)* containing the experimental data for the samples forming part of the study; *iii)* containing the representation of the relationship between sample units and experimental data. Another class of fundamental tools are visualization and analysis tools. Table 1 summarizes the main characteristics of these resources. For a deeper list of tools, see Kannan L [34, 35].

**Table 1:** Multiple cancer data type visualization and/or integration resources

Name	Description	Data type	Software type/Programming language	Key task	Operating system	Latest update
Caleydo StratomeX [24]	Tools allowing exploration of relationship among multiple datasets	Multi-omic	Application/Java	Data visualization	Windows Unix/Linux Mac OS	2018
CAS-viewer [25]	Visualization of Cancer Alternatively Splicing (CAS) is a dynamic interface providing an integrated knowledge of alternative mRNA splicing patterns along with multi-cancer omic data from 33 TCGA cancer types	DNA methylation, miRNAs, and SNPs	Web Application/-	Data visualization and basic analysis	Windows Unix/Linux Mac OS	2018
cBio Cancer Genomic Portal [26, 27]	The cBioPortal for Cancer Genomics provides visualization, analysis and download of large-scale cancer genomics data sets from The Cancer Genome Atlas as well as many carefully curated published data sets	Transcriptomic, DNA methylation, CNVs, SNPs and clinical data	Web Application/Python, Java, Perl, R, MatLab	Data visualization and basic analysis	Windows Unix/Linux Mac OS	2018
Genboree Workbench [28]	Genboree is a web-based platform for multi-omic research and data analysis using the latest bioinformatics tools.	Transcriptomic and epigenomic data	Web Application/-	Data visualization and basic analysis	Windows Unix/Linux Mac OS	2014
MARIO [29]	MArkov Random fields to Integrate Omics variables (MARIO) is a hierarchical Bayesian model approach for the parallel, integrative analysis of data from several genomic types	Multi-omic and beyond	BUGS software/-	Data integration/analytics	Unix/Linux	2017
mixOmics [30]	mixOmics offers exploration and integration of biological data and allows multivariate statistical approaches to identify similarities between two heterogeneous datasets	Multi-omic and beyond	Bioconductor Package/R	Data integration/analytics	Windows Unix/Linux Mac OS	2018
ModulOmics [31]	ModulOmics identifies cancer driver pathways, or modules, by integrating multiple data types on the basis of DNA and RNA cancer patient data, integrated with PPI networks and known regulatory connections	Multi-omic and beyond	Package/R or Python	Data integration/analytics	Unix/Linux Mac OS	2018
Omics Integrator [32]	Omics Integrator provides integration of proteomic data, gene expression data and/or epigenetic data using a protein–protein interaction network. It is comprised of two modules, Garnet and Forest	Multi-omic and beyond	Package/Python	Data integration/analytics	Unix/Linux	2018
XENA UCSC browser [33]	It offers interactive visualization and exploration of TCGA genomic, phenotypic, and clinical data, as produced by the Cancer Genome Atlas Research Network	Multi-omic	Web client	Data visualization	Windows Unix/Linux Mac OS	2017

### Challenges of radiomics in multi-omics framework

A crucial aspect in radiogenomic data analysis is the very large dimensionality of the feature space; therefore the analyses of these data are often unreliable and have a high overfitting and curse of dimensionality . For these reasons, radiogenomic studies need a robust data structure in order to reduce difficulty and make the analyses efficient, scalable and reproducible. In this context, some data integration and data processing challenges need to be addressed [36]. One challenge regards data acquisition of ‘omic experiments, since biological processes may be assessed in different spatial and/or temporal scales. Indeed there is a greater complexity in some type of ‘omics, such as transcriptomics (alteration of gene expression over time), compared to, for example, genomic experiments (executed on a temporal/spatial static substrate) [37]. Obviously, in radiogenomic data integration, the different and specific spatial/temporal multi-dimensionality introduces an additional level of complexity. For instance, in patient with cancer, imaging is usually performed multiple times during the course of disease and therapy whereas only one time and at one location genomics or transcriptomics profiling is not performed systematically [38]. Another crucial aspect in radiogenomic data analysis is the management of multi-sample and multi-parameters storage from different lesions or sub-regions of a lesion, for each patient. Spatial multi-dimensionality is a common event for both radiomic and biological ‘omics but, here too, there are cases of uniqueness. For example, in a proteomic experiment, which analyzes the abundance of proteins, their post-translational modifications and subcellular compartments location, does not have a  corresponding dimensionality in radiomic experiments. Another intrinsic problem of multi-omics analyses is missing data, which may occur due to reasons such as data filtering (for example, low coverage of a detected variant) or non-execution of a specific analysis on a subset of samples deriving from different laboratory. Different machine learning approaches are used to handling missing data [39] but a preliminary overview and quantification of these data is crucial to set a multi-omics analysis. Therefore, from a data structure point of view, the challenge is to ensure structured relations between patient data and experiments/assays features. One way to ensure the alignment of data is to take into account: the different scales of dimensionality of heterogenous data, missing data and data storage. Despite the challenges and the limitations described above, one of the biggest advantages of radiogenomic studies is the opportunity to assess the relationships between genotype features (such as genomic variants), intermediate phenotype features (such as transcriptomics and epigenetic variables), radiomic features (image phenotype) and phenotypic clinical outcome. Adding radiomic features means adding phenotypic descriptors, which differ from phenotypic outcome, but in relation with them and with the multi-omic biological features.

## Role of radiogenomics in cancer phenotype definition

Radiogenomic analyses are generally used for two main purposes: identifying features that might be related to genetic or molecular outcomes and correlating imaging and genomic data to identify suitable markers or predictors of a particular disease. Tipically, a radiogenomic dataset contains genomic (for example, gene expression) and imaging data, without outcomes data. A well-known correlation between specific imaging features and an outcome could enable the discovery of relationship between those features and specific tumor molecular characteristics.  Similarly, investigating a well-known correlation of tumor molecular characteristics related to an outcome may allow the detection of imaging features related with that outcome. Several studies, based on these approaches, have been published. Gevaert et al. [40] tested how well the imaging features, based on specific genomic characteristics, predicted patient survival in non–small-cell lung cancer using sets of imaging and genomic (gene expression) data without outcomes. Other examples are radiogenomic studies in which correlations have been detected between imaging features and tumor subtypes, especially in breast cancer and in glioblastoma multiforme. Mazurowski et al. [41] demonstrated that imaging features describing tumor enhancement dynamics can differentiate breast cancer luminal B molecular subtype from other subtypes. Therefore, an imaging feature might be predictive of outcomes and might not necessitate a genomic analysis. However, in another study, Guo et al. [42] describe that a combination of imaging and genomic features could be useful for better breast tumor characterization. Indeed, they demonstrated that imaging features such as tumor size outperformed genomic features in predicting tumor pathological stage, whereas genomic features outperformed imaging features in predicting breast cancer estrogen receptor (ER) and progesterone receptor (PR) status such as tumor molecular characteristics. Another approach was used in Karlo et al. [43], where correlations between imaging features and mutation of genes (related with stage and diminished survival prognosis) were identified. Through this evidence, imaging features, potentially predictive of outcomes, have been identified. Furthermore, in Glioblastoma Multiforme, 1p/19q co-deletion, a widely used prognostic biomarker for brain tumors, and epidermal growth factor receptor (EGFR) mutations, have been correlated with a wide array of MRI features [44, 45]. Finally, radiogenomics could potentially have an important role in targeted therapies and in improving the performance in cancer outcomes prediction. In order to identify complex phenotypes from a radiogenomics approach, a number of challenges need to be addressed. The introduction of more complex models combining multiple heterogeneous data sources could overcome many of these challenges. For further insights on state of the art of radiogenomics studies see [6, 46, 47].

## Radiomics in multi-omics framework: limits, challenges and limitations

### Existing integrated databases

Integrated databases share data across multiple data types ranging from clinical to ‘omics and medical imaging relative to specific research area. In Table 2, we provide a list of discipline-specific databases covering oncological, neurological, neurodegenerative and cardiovascular field or multiple-diseases. So far, multi-omic profiles are primarily available in the oncological field. Indeed, Genomic Data Commons (GDC) portal, which includes the TCGA database, and TCIA are an unprecedented source of biomedical data for a broad range of cancer diseases. Although each database possesses its own organization, overall, they store data sets with multiple data types available at different levels. In addition to multi-omic and imaging data, supporting data related to the images such as patient outcomes, treatment details, genomics, pathology, and expert analyses are also provided when available. Clinical, multi-omic and pathological data stored on the GDC can be associated to the imaging data, stored on TCIA. Although both data portals allow an interactive navigation through different projects and their multiple data types, using matched TCGA patient identifiers, it is possible to explore the TCGA/TCIA databases without the ability to automatically correlate tissue genotype, radiological phenotype and patient outcomes. For example, many TCGA/TCIA studies [70–72] have published their radiomic data (radiomic features, radiologist features or also segmentations) on the TCIA website. These data are in a simple table format, such as xls format, and at present there is no way to automatically explore the radiomic data together with the genome data available on TCGA portal. The current workflow consists of downloading imaging and genomic features separately, integrating the data through a non standard way and finally performing a cleaning and subsetting operation. The results of this process are likely to result in a situation in which there is either little or no suitable omic data. Thus, there is an urgent need to link radiomic and genomic data globally such that data integration in achieved, facilitating scientists to uncover genotype–phenotype associations/correlations. In the following section, we provide a case study based on breast cancer data from TCGA/TCIA database to illustrate an example of data integration and utilization of MAE data structure for multi-omics data management.

**Table 2:** Integrated Database of oncological, neurological/neurodegenerative, cardiovascular and multiple diseases

Name	Description	Data type	Data access	Data download	Latest update
Oncological disease					
CPTAC [48] https://cptac-data-portal.georgetown.edu/cptacPublic/	The Data Portal represents the NCI’s largest public repository of proteogenomic comprehensive sequence datasets	MS proteomic and phosphoproteomic data and gene expression	Open/controlled user account (open use studies) or request access by data use application (controlled use studies)	Web based, web client and programmatic	2018
GDC [49] https://portal.gdc.cancer.gov/	The Cancer Genome Atlas is a large cancer genomics data collection covering 43 projects with normal-control. Patient outcomes, treatment details, pathology, and expert analyses are also provided when available. Many subjects possess corresponding imaging data on The Cancer Imaging Archive (TCIA)	Gene expression, DNA methylation, germline and somatic mutations, clinical data	Open/controlled user account (open use studies) or request access by data use application (controlled use studies)	Web-based, web client and Programmatic	2018
ICGC [50] https://dcc.icgc.org/	The International Cancer Genome Consortium archives large number of datasets with molecular data from more than 20,000 donors including the Pan cancer Analysis of Whole Genomes (PCAWG) study	Germline and somatic mutations, gene expression, DNA methylation	Open/controlled user account (open use studies) or request access by data use application (controlled use studies)	Web based, web client and programmatic	2018
TCIA [51] http://https://www.cancerimagingarchive.net/	The Cancer Imaging Archive collects medical cancer images accessible for public download. Data include 78 collections and different image modalities. Many subjects possess corresponding genomics data on the GDC (ex TCGA)	Medical images in DICOM format, clinical data	Open/controlled user account (open use studies) or request access by data use application (controlled use studies)	Web based, web client and Programmatic	2018
Neurological and neurodegenerative disorders					
1000 Functional Connectomes Project/INDI International NeuroImaging Data-sharing Initiative [52] and curse of dimensionality [4]. https://www.nitrc.org/projects/fcon_1000/	It provides the broader imaging community complete access to a large-scale functional imaging dataset such as prospective, retrospective dataset	Imaging and clinical data	NITRC account for some public datasets and some controlled dataset	Amazon Web Services S3 and CyberDuke web client and command line	2018
LONI Database (The Laboratory of Neuroimaging at University of Southern California) [53] https://loni.usc.edu/about_loni	Repository for sharing and long-term preservation of neuroimaging and biomedical research data especially on neurological, neurodegenerative and psychiatric diseases. Some studies ongoing are: ADNI, ENIGMA, GAAIN, PPMI	Clinical, imaging (MRI, PET, MRA, DTI and other imaging modalities), genetic and behavioral data from multisite longitudinal study	Open use data required account controlled access by Image and Data Archive (IDA) request otherwise data use application request	Web-based Image and Data Archive (IDA)*	2018
LRRK2 Cohort consortium (The Michael J. Fox Foundation (MJFF) for Parkinson’s Research) [54] https://https://www.michaeljfox.org/page.html?lrrk2-cohort-consortium	The LRRK2 Cohort Consortium (LCC) comprises three closed studies: the LRRK2 Cross-sectional Study, LRRK2 Longitudinal Study and the 23 and Me Blood Collection Study	Clinical data and biospecimens (blood, urine and cerebrospinal fluid) from PD and control volunteers	Account controlled access data	LONI (IDA) repository^a^	2018
National Institute of Neurological Disorders and Stroke/The Michael J. Fox Foundation (MJFF) for Parkinson’s Research BioFIND [55] http://biofind.loni.usc.edu/	BioFIND is a cross-sectional clinical study designed to discovery new Parkinson’s disease biomarker	Clinical data and biospecimens (blood, urine and cerebrospinal fluid) from PD and control volunteers	Account controlled access data	LONI (IDA) repository*	2018
The National Institute of Mental Health (NIMH)/NIMH Repository and Genomic resources (RGR) [56] https://https://www.nimhgenetics.org/ http://ndar.nih.gov/	The NIMH Repository is an infrastructure for sharing data collected by hundreds of research projects in concerns clinical and genetic analysis of mental health disorders (e.g. schizophrenia, bipolar disorder, depression, Alzheimer’s disease, autism, obsessive–compulsive disorder, etc.). For instance the National Database for Autism Research (NDAR) website is the primary point of entry for Autism Research	Imaging Genetic and Clinical data	NIMH account approval	Web-based and web client Open Database License (ODbL)	2018
The National Institute of Neurological Disorders and Stroke (NINDS) [57] https://https://www.ninds.nih.gov/, https://pdbp.ninds.nih.gov/	The NINDS is divided into basic, clinical and translational research projects to advance the study of neurological disorders to both academic and industry investigators. One dataset is the PDBP DMR Parkinson’s Disease Biomarkers Program Data Management Resource	Gene expression, clinical data	NINDS account approval	Web-based and web-client Open Database License (ODbL)	2018
The National Institute on Aging (NIA)/AMP-AD Knowledge Portal Accelerating Medicines Partnership-Alzheimer’s Disease [58] https://http://www.synapse.org/#!Synapse:syn2580853/wiki/409840	The AMP-AD Knowledge Portal is the NIA-designated repository for distribution of data from multiple NIA-supported programs on Alzheimer’s disease	Various types of molecular data from human, cell-based and animal model biosamples	Account controlled access data	Synapse web browser and web client	2018
The National Institute on Aging Genetics of Alzheimer’s Disease (Data Storage Site NIAGADS) [59] https://http://www.niagads.org/	The NIAGADS provides access to publicly available NIAGADS summary statistics datasets for Alzheimer’s Disease and related neuropathologies	Multi-omic GWAS, whole genome (WGS) and whole exome (WES), expression, RNA Seq, and CHIP Seq analyses	Open to investigators return secondary analysis data to the database	Web-based (NIAGADS genome browser) and web-client Open Database License (ODbL)	2018
Cardiovascular disease					
Cardiac Atlas Project [60] http://http://www.cardiacatlas.org/	A multi-center cardiac MRI data sets with the most robust manual contours defined by the consensus of 7 independent expert readers from 7 world-class core labs. Datasets related to 6 different studies	Imaging (MRI data) and clinical data	Controlled CAP data access request	Web client	2018
National Heart, Lung, and Blood Institute (BioLINCC) [61] https://biolincc.nhlbi.nih.gov/home/	NHLBI is the NIH center devoted to research, training, and education of heart, lung, blood and sleep disorders. It provides teaching datasets and public use datasets	Clinical data and sometimes corresponding biospecimens	Open and controlled data on request	Web-based user interface (BioLINCC)	2018
The Cardiovascular Research Grid (CVRG) [62] http://cvrgrid.org/	The CardioVascular Research Grid (CVRG) project is supported by the National Heart Lung & Blood Institute for creating an infrastructure for sharing cardiovascular data and data analysis tools	Imaging (ex vivo DWI and in vivo heart CT) and clinical data	Open/Controlled	Web-based	2018
The Qatar Cardiovascular Biorepository (QCBio) [63] http://http://www.qcbio.org/	Cases include patients needing percutaneous intervention for symptomatic coronary heart disease (CHD) or admitted with an acute coronary syndrome (myocardial infarction or unstable angina). Controls are individuals identified from the Hamad Medical Corp. blood bank who have no history of CHD. The goal of QCBio is to archive plasma and DNA of 1000 Qatari patients with coronary heart disease and 1000 controls, who are matched on age, sex and ethnicity	Biospecimens (plasma and DNA) and clinical data	Open to Qatari investigators and controlled access data for others	Web-based and web client	2018
Vascular Diseases Biorepository [63] https://http://www.mayo.edu/research/labs/atherosclerosis-lipid-genomics/research-projects/vascular-diseases-biorepository	Biorepository for common vascular diseases, including: (PAD) Peripheral artery disease, aortic aneurysm, (CAD) carotid artery stenosis, fibromuscular dysplasia. These samples are linked with demographic information, conventional cardiovascular risk factors, and comorbidities ascertained from Mayo Clinic’s electronic health record using EHR-based electronic phenotyping algorithms	Biospecimens (DNA, serum and plasma) and clinical data	Open/controlled	Web-based and web client	2018
Multiple diseases					
DAA [64] http://ageing-map.org/atlas/	The Digital Aging Data is a portal of age-related changes covering different biological levels. It integrates to create an interactive portal that serves as the first centralised collection of human ageing changes and pathologies	Gene expression and proteomic, psychological and pathological age-related data	Publicly available by DAA account approval	DAA account approval for open	2017
dbGaP [65] https://https://www.ncbi.nlm.nih.gov/gap/	The database of Genotypes and Phenotypes (dbGaP) was developed to archive and distribute the data and results from studies that have investigated the interaction of genotype and phenotype in Humans. Over 150 NCI studies are registered in dbGaP	Genome wide studies and clinical data	Open/controlled NCBI account approval	Web client and programmatic	2018
EGA [66] https://https://www.ebi.ac.uk/ega/	The European Genome-phenome Archive collects human biomedical data across Europe. It allows authorised users to search sequenced material, patient samples stored in biobanks, patients illnesses, treatments, outcomes	Imaging Gene expression, genome wide studies and clinical data	Controlled data use application request, then EGA account approval	Web client and programmatic	2018
GEO [67] https://https://www.ncbi.nlm.nih.gov/geo	Gene Expression Omnibus provides multiple level datasets (4348 in total) related to cancer and other diseases	Gene expression, genome wide studies and clinical data	Most data are publicly available, sometimes data use on request	Web client and programmatic	2018
HGAR [68] http://genomics.senescence.info/	The Human Ageing Genomic Resources (HAGR) is a collection of databases and tools designed to help researchers study the genetics of human ageing using modern approaches such as functional genomics, network analyses, systems biology and evolutionary analyses	Gene expression and clinical data	Publicly available raw data, processed data on request	Web based download (zip, csv files)	2018
JGA [69] https://https://www.ddbj.nig.ac.jp/jga/index-e.html	Japanese Genotype-phenome Archive is a service for archiving and sharing of all types of individual-level genetic and de-identified phenotypic data	Imaging, gene expression, genome wide studies and clinical data	NBDC Human Database approval	Web client and programmatic	2018

^a^LONI (IDA) repository of multiple projects

### Statistical challenges

The increasing interest in the development of statistical methodologies for multi-layers integration is due to the complexity of biological systems and data heterogeneity. In particular, to integrate heterogeneous data several methodological challenges must be addressed must:

1. different technical platforms;
2. different modalities and techniques used to acquire and measure data;
3. different numerical data types and scales;
4. large differences in the number of measured features for each data type.

In a multi-assay context, these factors make it difficult to choose the appropriate statistical approaches for data processing and the integration method. Each technical platform has its own noise level and sensitivity and, generally, it is associated with ad-hoc protocols for normalization and batch effects, depending on ‘omics/radiomics data type. Heterogeneous data integration includes the following statistical issues: dimension reduction, data integration or data fusion and causal inference:

#### Dimension reduction

In multi-assays integration context, heterogeneous data usually increase the dimensionality and, consequently, increase the chance to produce false positive hypothesis testing results. To solve this problem, the first step is to identify and combine relevant features from each data modality, keeping known the biological dependencies. Dimension reduction approaches decompose data into a few new variables (called components) that explain most of the differences in observations. Dimension reduction approaches, widely used in exploratory analysis of single omics datasets, are emerging also to simultaneous exploratory analyses of multiple datasets. These methods extract the linear relationships that better explain the correlated structure across datasets, the variability both within and between variables (or observations) and may highlight data issues such as batch effects or outliers. In the literature for integrated ‘omics, dimension reduction methods have presented several variations from Principal Component Analysis (PCA) and Factor Analysis. These variations include Multiple Factor Analysis (MFA), consensus PCA (CPCA), multiple-block PCA (MBPCA) and non-negative matrix factorization (NMF). As ‘omics datasets tend to have high dimensionality it is often useful to reduce the number of variables. In fact, several recent extensions of PCA include variable selection, often via a regularization step or L1 penalization (e.g. Least Absolute Shrinkage and Selection Operator, LASSO).

#### Data integration or data fusion

Two main approaches to multi-omics data integration can be considered: linear or simultaneous integration. The linear approach to multi-omics data leads to an oversimplified view of biology, basing on already known biological processes. This is possible, in particular, when only two data types are considered. The complexity of the phenotypes suggests that they can be better explored by the combination of simultaneous changes across all ‘omics data. The linear multi-omics integration does not consider unknown inter-omics relationships. Instead, simultaneous approach provides a complete and realistic characterization of phenotype from exploring the inter-omics interactions. Statistical methodologies for simultaneous integration can be classified into supervised and unsupervised approaches. Unsupervised methods explore biological profiles from input datasets and assign objects into different subgroups (clusters) without labeled response variables. Conversely, supervised methods consider the available known phenotype information from samples (for example disease-normal, treatment–control) and use this information to discover genotype–phenotype interactions and investigate biological processes. In multi-omics data integration field, there are different statistical approaches that can be classified as multivariate, concatenation-based and transformation-based methods. Multivariate methods are usually based on Partial Least Square Regression (PLS) or Canonical Correspondence Analysis (CCA). Many of them were developed and integrated in multi-omics bioinformatics tools (Table 1). Concatenation-based integration methods are performed by combining multiple data matrices of different multi-omics data types into a single combined matrix, used as input for constructing a predictive model. Finally, the transformation-based methods, such as Similarity Network Fusion, before constructing a model, convert multi-omics data types into intermediate and common form and integrate them into a large input matrix. The main advantage of a transformation step is to preserve individual ‘omics characteristics that can be lost otherwise.

#### Causal inference

A vital piece in understanding of the disease mechanisms. In genomic data analysis, we can consider different types of associations, such as association of discrete variables (DNA variations) with continuous variables (phenotypes, gene expression), association of discrete variables (DNA variations) with binary trait (disease status). In the integrated ‘omics literature, the regression strategies are used for explaining inter- or intra-system relations and interactions. One of the approaches is the parallel regression, used to explain intersystem responses simultaneously. Another possible approach is represented by Bayesian networks (BNs), belonging to the family of graphical models. BNs maintain high interpretability via graphical outputs and represent a way to identify possible causal relationships between measured variables depending on their conditional dependencies and independence. BNs explicitly model conditional statistical dependencies among random variables. In the biological context, each random variable represents one molecular feature. Integration of different ‘omics data modalities can be performed by using a primary data source, for example gene expression and employing further data (i.e., histone modifications or combinations of several sources) to construct informative network priors, which facilitate the identification of the true biological network from data. Networks represent a powerful tool in the context of multi-omics data integration, since they are able to contain heterogeneous and high-dimensional information. Networks can characterize complex interactions, thus identifying the mechanism linked to different types of information and associated to the phenotype of interest. In radiogenomics, a weighted network fusion that takes into account the importance of each layer could be considered. This approach can be applied to multi-omic genome-scale models where layers represent transcriptomic and phenotypic information. The weight measures the relative importance of each layer. Then each condition is associated with a point in a multi-dimensional phenotypic space. In order to address knowledge from the dynamic nature of molecular networks under various disease conditions, an unsupervised method, called DIABLO [73], was developed. DIABLO is an integrative classification method building predictive multi-omics models that can be applied to multi-omics data from new samples to determine their phenotype. This approach includes sparse generalized canonical correlation analysis (sGCCA) [74], multi-omics factor analysis (MOFA) [75], and Joint and Individual Variation Explained (JIVE) [76]. The latter is a component-based method: it transforms each ‘omic dataset into latent components and maximizes the sum of pairwise correlations between latent components and a phenotype of interest.

## MAE framework design: a case study

As described in the previous sections, we propose the use of MultiAssayExperiment (MAE) object as data structure to integrate genomic, radiomic and clinical data, providing coordinated representation, operations on multiple and heterogeneous data and focusing on two fundamental aspects of data at stake: multisampling and data longitudinality. We tested this solution at first studying existing MAE objects of TCGA unrestricted data of different cancer tissue obtained through curatedTCGA R package [77] and then creating a new MAE based on the TCGA breast cancer data and the respective radiomic features, extracted from T1 weighted Dynamic Contrast Enhanced (DCE) MRI images of TCIA [71].

**Fig. 2:**
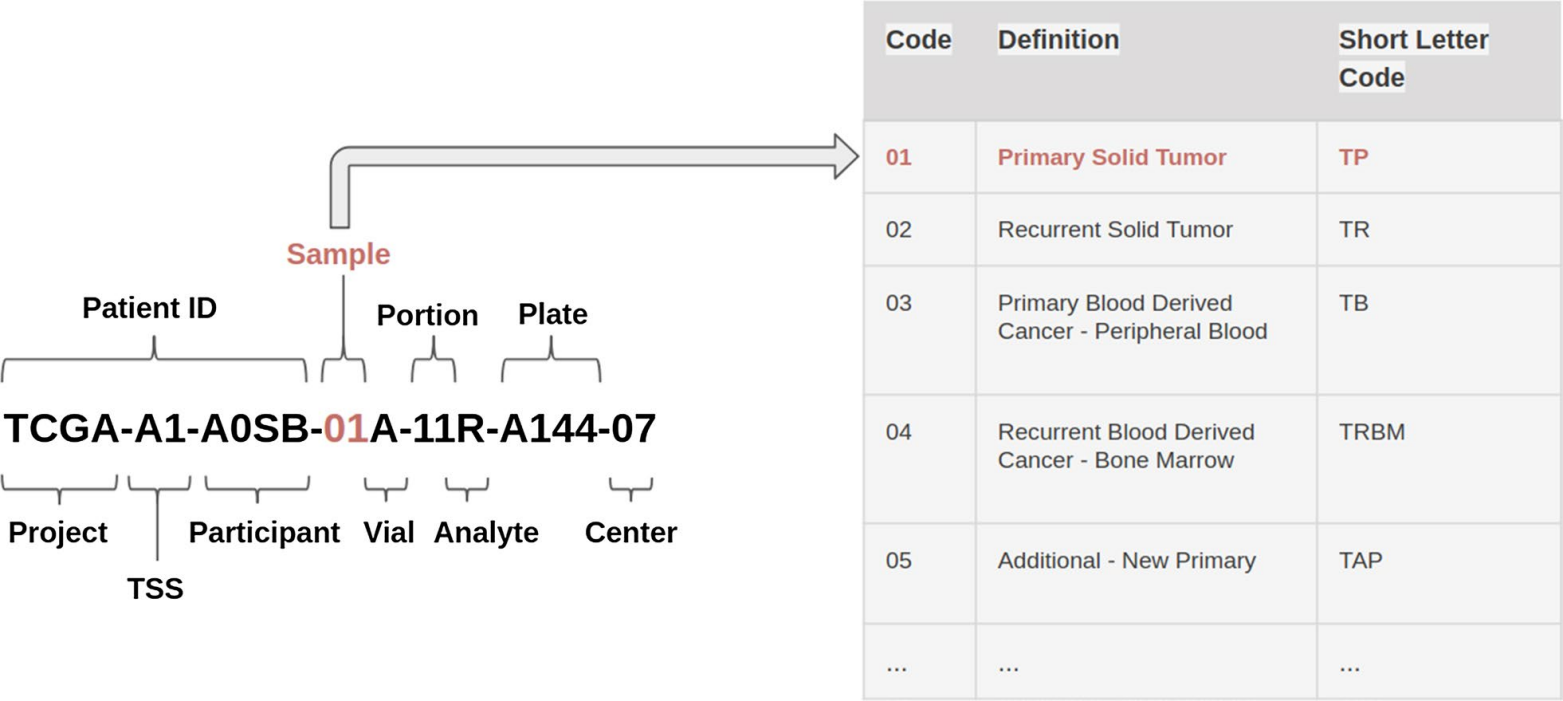
A barcode example. An example of a The Cancer Genome Atlas barcode with a focus on the Sample Type Codes table. Some of the identifiers, such as Vial, Portion, Analyte and Plate, are specific for biological experiments and obviously are not usable for radiomic experiments

**Fig. 3:**
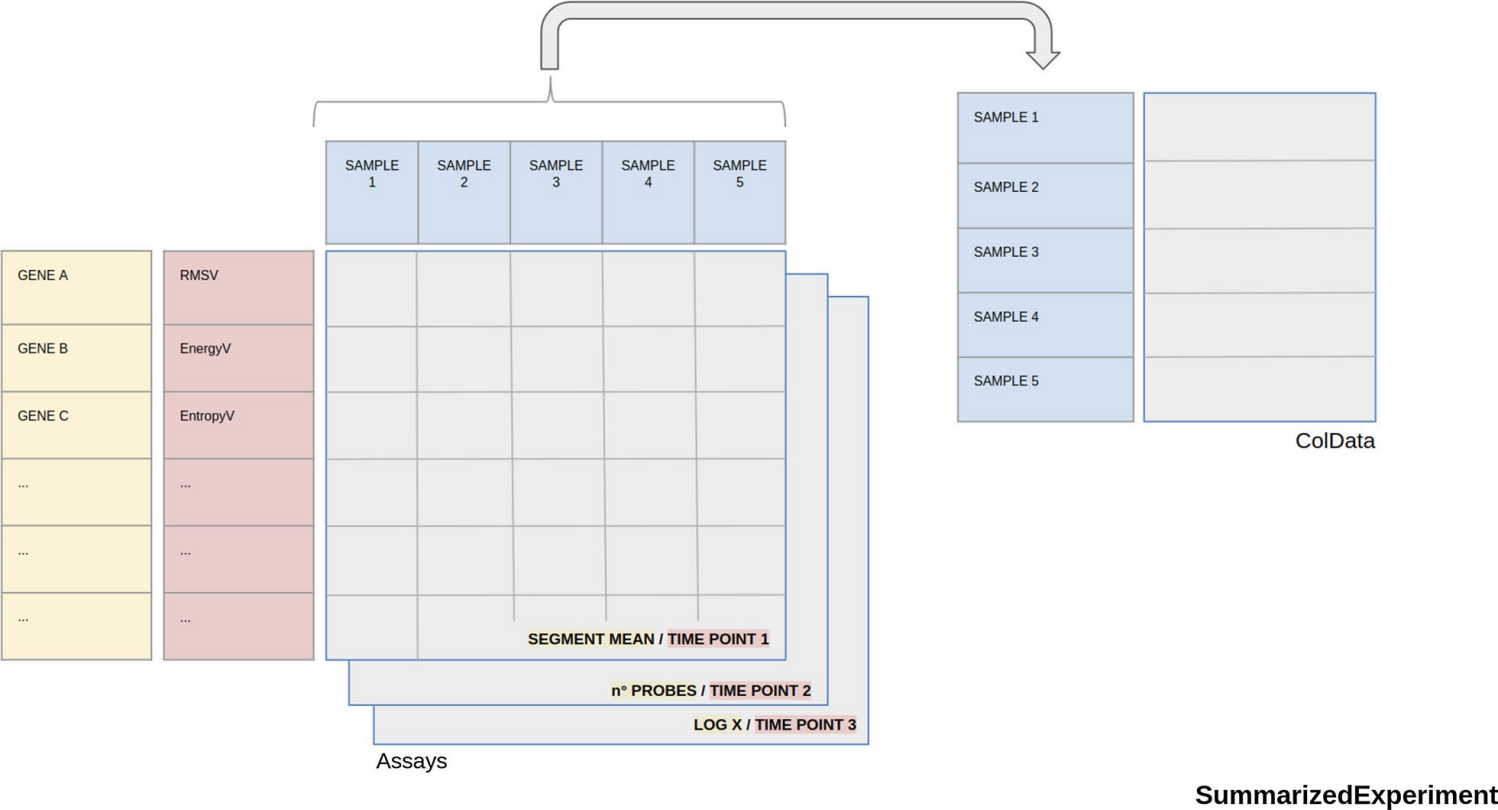
SummarizedExperiment object schema. In yellow: a classic use of summarizedExperiment object to store biological ‘omic experiment data. Each assay contains data for a result of the experiment (in this case segment mean, no probes and Log X from a Copy Number Alterations experiment). The rows of SE represent the genes and the columns represent the samples. Data describing the samples are stored in ColData object. In red: a summarizedExperiment with Magnetic Resonance Time Points as different assays. Each assay of the summarizedExperiment contains data of a single time-point and the rows represent radiomic features

**Fig. 4:**
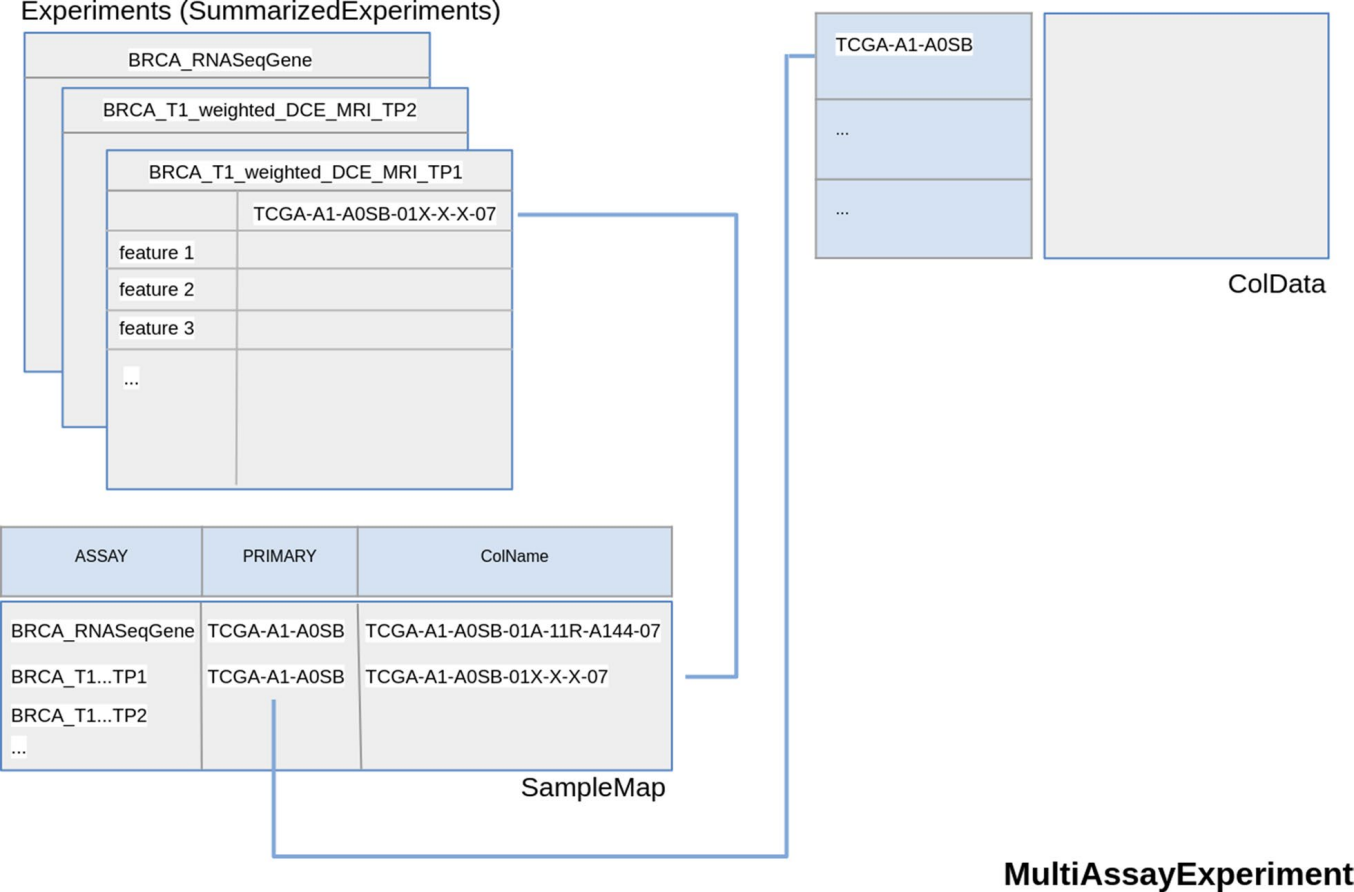
MultiAssayExperiment object schema with Magnetic Resonance Time Points as different Experiments. The second option described to store temporal multi-dimensionality of a radiomic experiment. Each element of Experiments (in this case a SummarizedExperiments) object of the MultiAssayExperiment contains data of a single time-point. TRhe radiomic features are also contained in the rows of SummarizedExperiment

Objects from curated TCGA contain data from different ‘omic experiments carried out on the same patient. Each experiment may contain different sample types for the same patient (for example, primary solid tumor and metastatic samples). To manage these data, in the TCGA project, a barcode is used as primary identifier. The TCGA barcode consists of a sequence of values associated to labels, each of which specifically identifies a TCGA data element. For example, the “Sample” label describes the sample type of a particular collection of data related to a patient and may take different value corresponding to a sample type according to Sample Type Codes table [78]. The barcode TCGA-A1-A0SB-01A example indicates Primary Solid Tumor data (Sample Type Code: 01) of the patient A0SB belonging to TCGA project on breast invasive carcinoma (Tissue Source Site Codes: A1) (Fig. 2). This nomenclature was also used by curated TCGA in the construction of the TCGA data MAE object. In more detail, the barcode is used as value of *colname* column of MAE *sampleMap* (a *DataFrame* that relates the “primary” data - that describes the biological unit, which can refer to specimens, patients, etc.—to the experimental assays—for example, RNAseqGene). This *DataFrame* allows an unambiguous map from every experimental observation to one and only one biological unit, such as a patient, and allows different technical and biological replication for each assay. Moreover, identifiers allow consistency between data during subsetting and re-ordering. We propose to use both MAE data structure and a TCGA-barcodes-like structure to manage radiomic experiment data, together with biological omic data, in a single data structure. Typically, in a radiomic workflow, each single ROI, and the respective features, may represent a different lesion, or a region of a lesion, and, therefore, may exist in multiple samples for each patient of a radiomic experiment. According to our proposal, this multi-sampling feature can be managed by using MAE data structure and a specific barcode, in the same way as genomic data. In this scenario, a key role is played by the *ExperimentList* component of MAE, which contains all experimental data. This component can contain different type of elements, two of which are *Matrix* (a base element used for ID-Based dataset) and *SummarizedExperiment* [79]. The latter may contain one or more assays (a matrix-like element that store the data). For each assay, rows represent features of interest and columns represent samples. For example in a genomic experiment, the rows of an assay represent genes or transcript, the columns represent the patients and each different assay in a Copy Number Alterations experiment may represent the values of segment mean and number of probes (Fig. 3). A radiomic experiment differs substantially from a genomic experiment in that it consists of one level of data complexity less than a genomic experiment. The latter is performed on a set of samples (the columns of the assay) and the analysis is performed on a set of genes, transcripts or protein (the rows of the assay). Finally, the experiment produces different results from the various assays. A radiomic experiment, instead, is performed on a set of samples without analysing of the molecular sub-level and therefore, from data structure point of view, we have two options:

- Use assays of a summarizedExperiment to store the matrix-like data of each time-point. In this case, multiple time-point data are associated to a single experiment, for example BRCA_T1_weighted_DCE_MRI, with as many assays as time-points (BRCA indicates breast cancer data) (Fig. 3).
- Use different summarizedExperiment to store different time-point data. In this case two experiments may be, for example, BRCA_T1_weighted_DCE_MRI_TP1 and BRCA_T1_weighted_DCE_MRI_TP2 (TP indicates Time Point) (Fig. 4).

In both cases, the rows of each assay stored radiomic features.

**Fig. 5:**
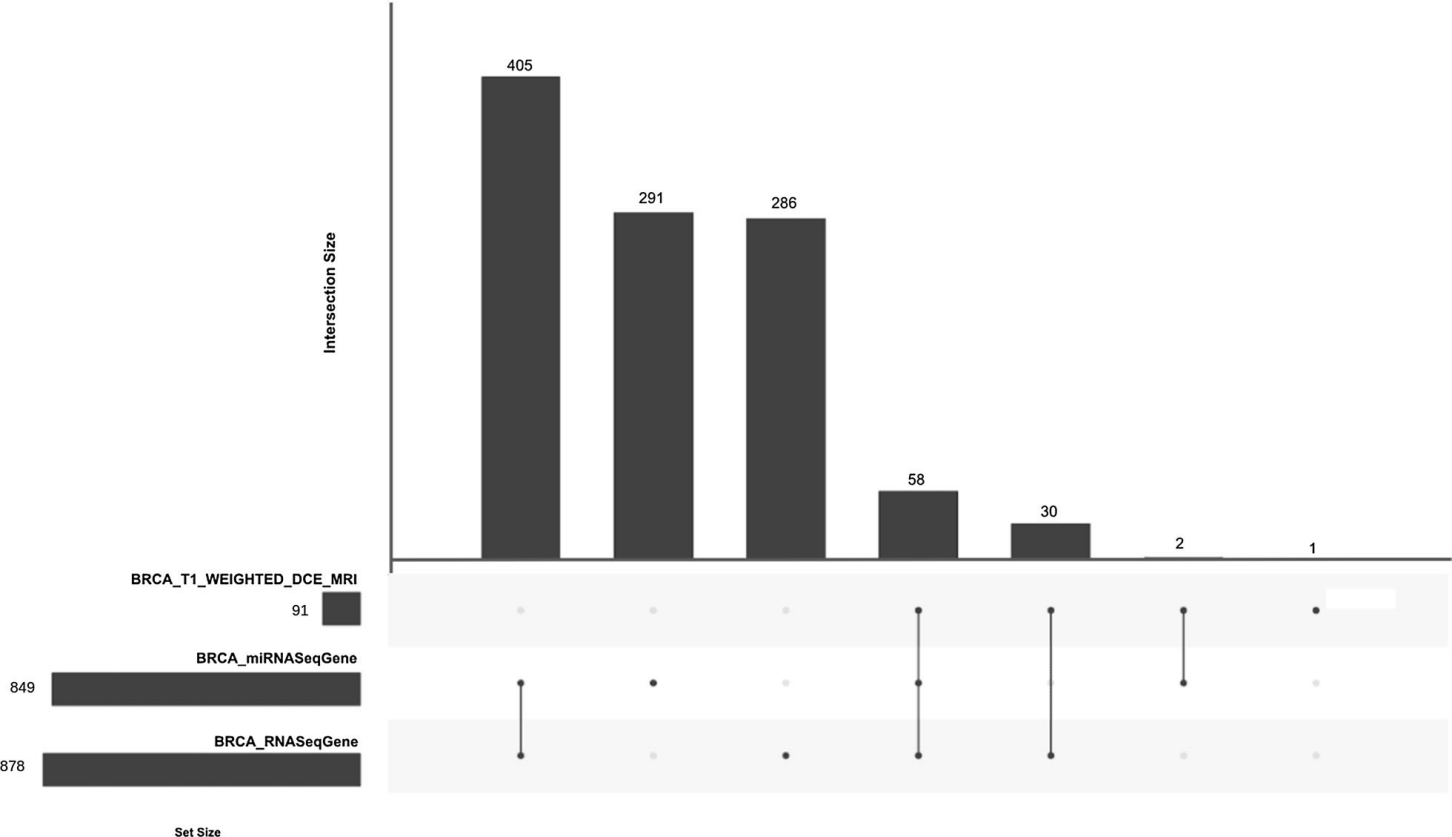
A generalized Venn diagram for sample membership in multiple assays. The visualization of set intersections was performed using the UpSet matrix design using UpSetR package

**Fig. 6:**
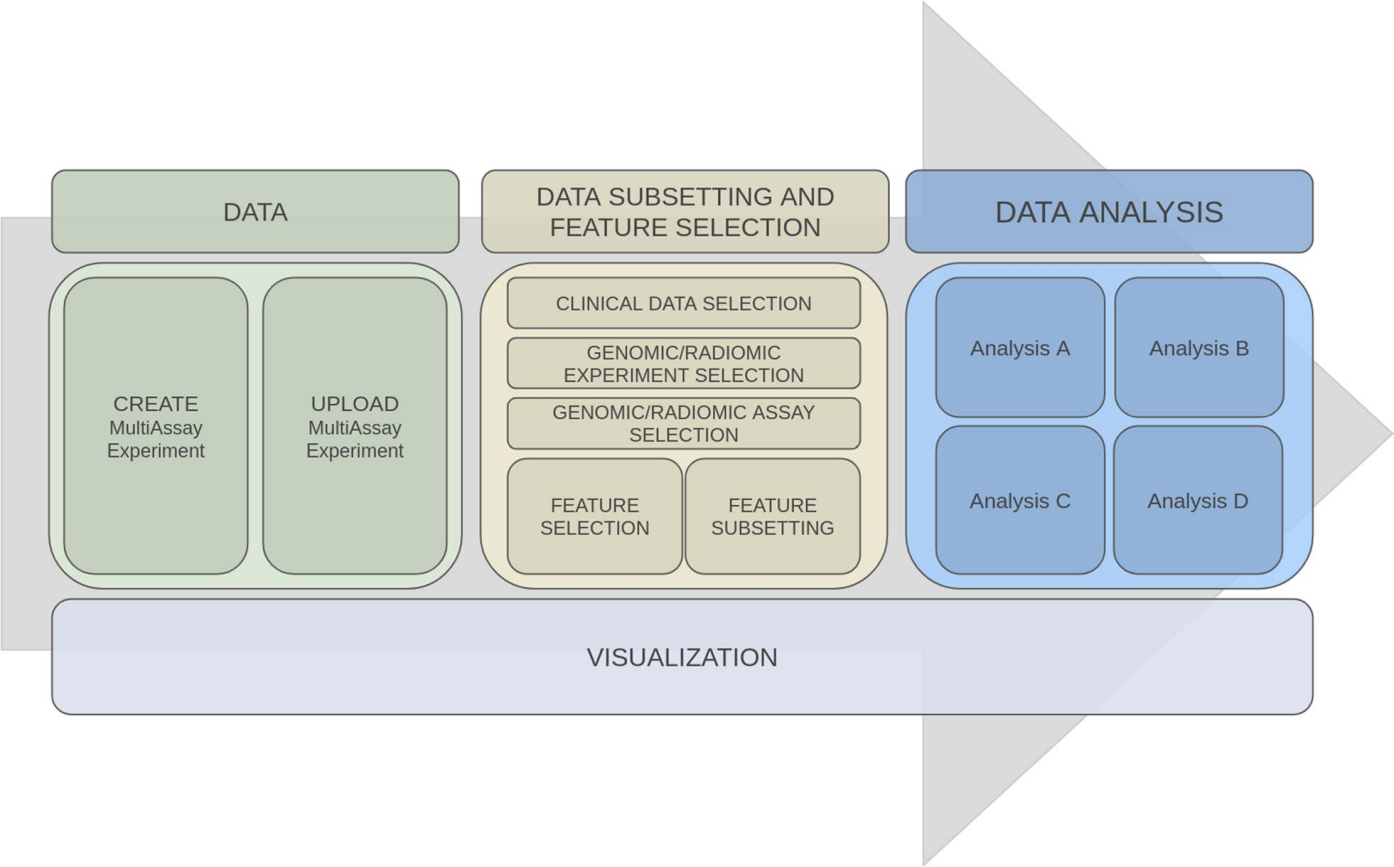
Architecture of the modular integration platform. The architecture herein proposed follows three separate modules. The first module, based on data uploading of a MultiAssayExperiment or from its construction from multiple SummarizedEXperiment or matrix-like data. The second module allows to execute different selections of data (by clinical data, such as pathological stage or histological type of cancer, by experiment/assay and features). Then selected data are the input of different and/or integrate data analysis module. This modular architecture simplify expansion and redesign of a single implementation and allow simple adding of a personal module of data preparation and/or analysis for specific tasks. Moreover, all modules may provide visualization of data to support the different operations (see an example of data visualization in Fig. 6)

**Fig. 7:**
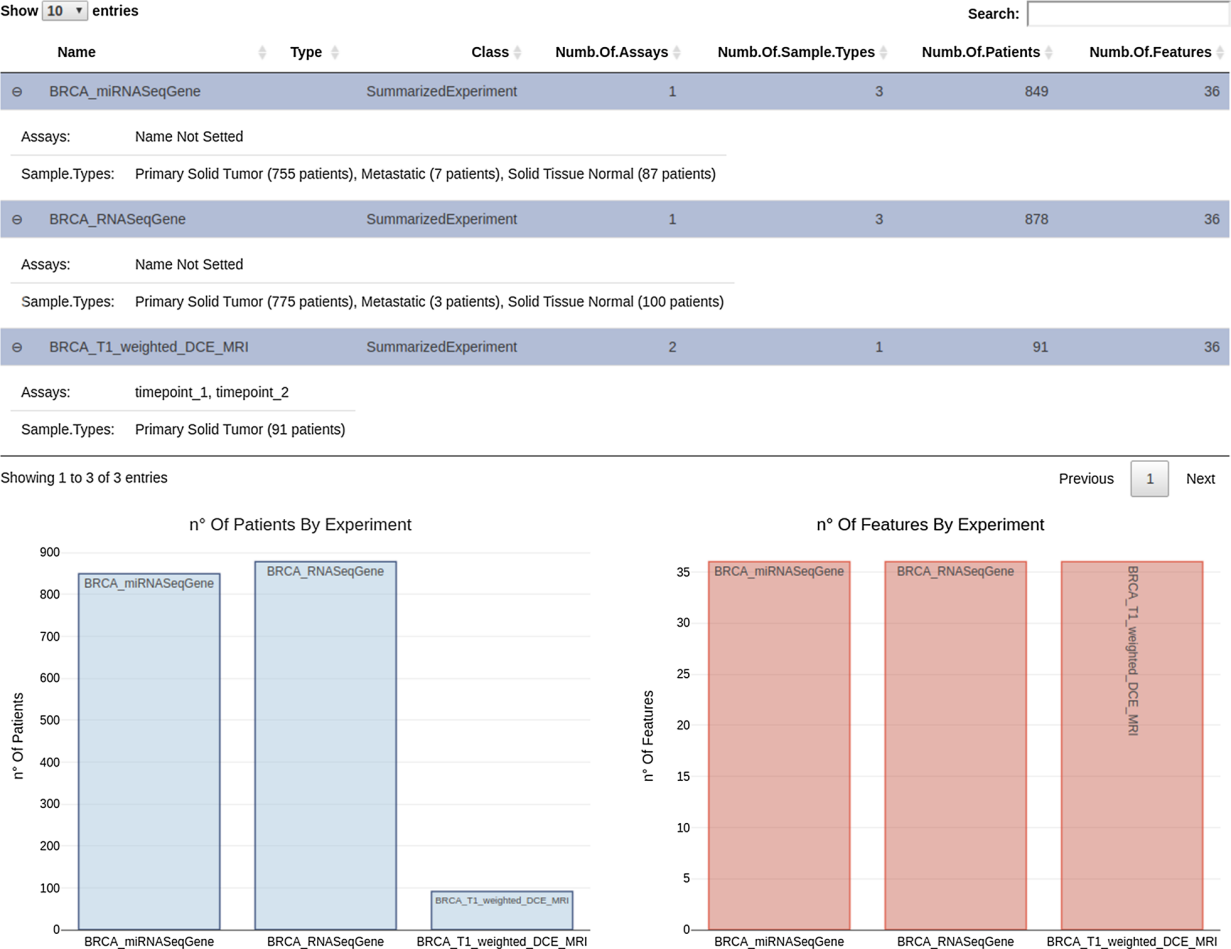
A screenshot of summary tab of the graphic interface prototype. The summary tab shows the MAE data of the described case study. In the top table the name of all MAE experiments are listed and for each of them are reported the assays (timepoint_1 and timepoint_2 in the case of BRCA_T1_weighted_DCE_MRI) and the sample types. For each sample type, the number of patients is specified. The number of features and patients for each experiment are also represented as histogram (for a simple graphic representation the number of features was limited to 36 for all experiments)

As shown in our case study, this data organization enables the use of MAE to collect, manage and then analyze radiomic data together with genomic and clinical data. In our case study the TCIA data consist of 36 quantitative radiomic features extracted from primary tumor images of 91 patients of the BRCA study, each acquired at a single time point. TCGA data consist of a MAE object composed of several experiments. We selected RNA-seq (expression quantification of 20,502 genes from 878 samples) and miRNA-seq (expression quantification of 1046 miRNA from 849 samples) experiments and integrated it together with TCIA data in a single MAE object. To do this, we first downloaded TCGA experiments, using curated TCGA, and TCIA features released. Subsequently, we created two R objects: one for TCGA and one for TCIA data. In the case of TCGA data we have directly obtained a MAE object through curated TCGA while in the case of TCIA, we first converted an xls file in an R dataframe and then in a summarizedExperiment object. This experiment contains two assays that represent two time point data, one of which is simulated. The radiomic experiment was subsequently integrated with the two pre-existing summarizedExperiment (RNA-seq and miRNA-seq experiment extracted from the downloaded MAE) using the workflow described in MAE vignettes on Bioconductor [80]. Figure 5 shows a generalized Venn diagram for sample membership in multiple assays. The visualization of set intersections was performed using the UpSet matrix design using UpSetR package [81]. The script code to reproduce above described procedure is available at https://gitlab.com/Zanfardino/radiogenomics-mae-case-study. We also propose an architecture, shown in Fig. 6, for a modular integration platform. Through a graphic interface, the users are able to (i) create or upload a MAE object, (ii) summarize MAE data through basic statistics and plots, (iii) manage and subset the uploaded data and (iv) execute different type of analyses (through independent modules). All the functions allow to work with highly complex data in an intuitive and simplified way. One way to manage and understand the meaning of large dimensional data is to place it in a visual context such as we have done here (Fig. 7).

## Conclusions

The high-throughput production of ‘omics data has led to an increase of data sets of different types that need to be integrated in order to better understand disease mechanisms and how these multiple molecular data generate the observed phenotypes in complex diseases. Merging imaging phenotypes with multi-omic biological data may lead to new prognostic cancer  models, new support for patient treatment strategy and development of improved survival predictors. Accordingly, increased attention is paid to statistical methods and algorithms to analyze and correlate multivariate imaging, clinical and molecular data for disease diagnosis and prognosis. Bringing these datasets together in a meaningful manner is the main goal of this study. Here, we identified three main challenges to overcome: the management of missing data caused by data filtering or non-execution of a specific analysis on a subset of samples, different spatial and temporal scales of imaging data and the need to manage radiomic features related to multiple lesions or sub-regions of a lesion. Our proposal to use MAE as data structure to combine radiogenomic data aims to integrate and facilitate the use and the exploration of heterogeneous and complex data derived from these deeply distant domains. Our integrated design enables regular operations of MAE on all experiments of a radiogenomic dataset, including radiomic data. With our MAE design, operations like: (i) selecting complete cases or subsettings, (ii) selecting samples with information in all dataset and/or in all ‘omics of a set of experiments (crucial to set a multi-omics analysis when more samples with missing data exist) and (iii) selecting subjects with specific phenotypes and clinical outcomes, may be carried out in order to ensure correct alignment of assays and patients, making radiogenomic integrative analysis more attainable. Moreover, the MAE structure, and the ability to organize data of different experiments in different interlinked data objects, has facilitated the handling of the additional spatial and temporal scales added by radiomic data. All of these operations and, therefore, our selection of the MultiAssayExperiment as the radiogenomic data container have been successfully tested with the integration of TCGA-BRCA data of 91 patients with radiomic features available on TCIA for the same patients. Despite the existence of specific software that make some of these functions available, such as TCGAbiolinks, RTCGA Toolbox and mixOmics (Table 1), none of these tools was designed to work on radiogenomic data. Conversely, our proposal facilitates radiogenomic studies since it allows user exploration across genomic as well as imaging datasets in data type independent manner. For example, the use of the TCGA barcode is clearly suitable for biological ‘omics data description but not for radiomic data, except for “Patient ID”, “Sample” and “Center” labels. The latter represents an open challenge and, therefore, a possible future direction might be the development of a unique nomenclature for a new type of barcode to specifically describe radiogenomic data. Moreover, for data different from TCGA, also a custom id can be used as colName in SampleMap. In conclusion, understanding the relationships among genomic profiles, imaging phenotypes and outcomes has great potential to improve cancer treatment and management. In this context, genomic features are closely related to genetic and molecular profile of a cancer and, consequently, to outcomes like receptor status, while, radiomic features characterize tumor phenotypes and, consequently, outcomes like tumor stage. Bringing radiomics and genomic data together into a single data structure is the first step to achieve effective radiogenomic analysis, integrating information arising from different aspects of the tumor. The use of the current version of MAE by an interdisciplinary research community can pave the way to further development of extended MAE object for implementing new functionalities specific to radiogenomic domain in order to correlate phenotype and genotype features.

## Data Availability

Data supporting the findings of this study are available from GDC data portal https://www.cancer.gov/about-nci/organization/ccg/research/structural-genomics/tcga and from TCIA web site http://doi.org/10.7937/K9/TCIA.2014.8SIPIY6G.

## Abbreviations

MAE: MultiAssayExperiment
NGS: next generation sequencing
TCGA: The Cancer Genome Atlas
TCIA: The Cancer Imaging Archive
DNA: deoxyribonucleic acid
ROI: region of interest
DW4TR: Data Warehouse for Translational Research
ER: estrogen receptor
PR: progesterone receptor
EGFR: epidermal growth factor receptor
GDC: genomic data commons
PCA: principal component analysis
MFA: multiple factor analysis
CPCA: consensus PCA
MBPCA: multiple-Block PCA
NMF: non-negative matrix factorization
LASSO: Least Absolute Shrinkage and Selection Operator
PLS: partial least square
CCA: Canonical Correspondence Analysis
BNs: Bayesian networks
sGCCA: sparse generalized canonical correlation analysis
MOFA: multi-omics factor analysis
JIVE: Joint and Individual Variation Explained
DCE: dynamic contrast enhanced
BRCA: breast related cancer antigens
MRI: magnetic resonance imaging
RNAseq: ribonucleic acid sequencing

## References

[1] Ramos M, Schiffer L, Re A (2017). Software for the integration of multiomics experiments in Bioconductor. Cancer Res.

[2] Hariri AR, Weinberger DR (2003). Imaging genomics. Br Med Bull.

[3] Baltrušaitis T, Ahuja C, Morency LP (2018). Multimodal machine learning: a survey and taxonomy. IEEE Trans Pattern Anal Mach Intell.

[4] Aiello M, Cavaliere C, D'Albore A (2019). The Challenges of Diagnostic Imaging in the Era of Big Data. J Clin Med.

[5] Gatenby RA, Grove O, Gillies RJ (2013). Quantitative imaging in cancer evolution and ecology. Radiology.

[6] Incoronato M, Aiello M, Infante T (2017). Radiogenomic analysis of oncological data: a technical survey. Int J Mol Sci.

[7] Gillies RJ, Anderson AR, Gatenby RA (2010). The biology underlying molecular imaging in oncology: from genome to anatome and back again. Clin Radiol.

[8] Aerts HJ, Velazquez ER, Leijenaar RT (2014). Decoding tumour phenotype by noninvasive imaging using a quantitative radiomics approach. Nat Commun.

[9] Diehn M, Nardini C, Wang DS (2008). Identification of noninvasive imaging surrogates for brain tumor gene-expression modules. Proc Natl Acad Sci USA.

[10] Segal E, Sirlin CB, Ooi C (2007). Decoding global gene expression programs in liver cancer by noninvasive imaging. Nat Biotechnol.

[11] Gillies RJ, Kinahan PE, Hricak H (2016). Radiomics: images are more than pictures, they are data. Radiology.

[12] Li H, Zhu Y, Burnside ES (2016). Quantitative MRI radiomics in the prediction of molecular classifications of breast cancer subtypes in the TCGA/TCIA data set. NPJ Breast Cancer.

[13] Yip SSF, Aerts HJWL (2016). Applications and limitations of radiomics. Phys Med Biol.

[14] Monti S, Aiello M, Incoronato M (2018). DCE-MRI pharmacokinetic-based phenotyping of invasive ductal carcinoma: a radiomic study for prediction of histological outcomes. Contrast Media Mol Imaging.

[15] Kirienko M, Cozzi L, Antunovic L (2017). Prediction of disease-free survival by the PET/CT radiomic signature in non small cell lung cancer patients undergoing surgery. Eur J Nucl Med Mol Imaging.

[16] Blanc-Durand P, Van Der Gucht A, Jreige M (2017). 18F-FDG PET-based radiomics score predicts survival in patients treated with Yttrium-90 transarterial radioembolization for unresectable hepatocellular carcinoma. J Nucl Med.

[17] Wang J, Wu CJ, Bao ML (2017). Machine learning-based analysis of MR radiomics can help to improve the diagnostic performance of PI-RADS v2 in clinically relevant prostate cancer. Eur Radiol.

[18] Jochems A, Hoebers F, De Ruysscher D (2017). Deep learning of radiomics features for survival prediction in NSCLC and Head and Neck carcinoma. Radiother Oncol.

[19] Ingrisch M, Schneider MJ, Nörenberg DN (2017). Radiomic analysis reveals prognostic information in T1-weighted baseline magnetic resonance imaging in patients with glioblastoma. Investig Radiol..

[20] Keek SA, Leijenaar RT, Jochems A (2018). A review on radiomics and the future of theranostics for patient selection in precision medicine. Br J Radiol.

[21] Huang S, Chaudhary K, Garmire LX (2017). More Is better: recent progress in multi-omics data integration methods. Front Genet.

[22] Hernandez-Ferrer C, Ruiz-Arenas C, Beltran-Gomila A (2017). MultiDataSet: an R package for encapsulating multiple data sets with application to omic data integration. BMC Bioinform.

[23] Hu H, Correll M, Kvecher L (2011). DW4TR: a Data Warehouse for Translational Research. J Biomed Inform.

[24] Streit M, Gratzl S, Stitz H (2018). Ordino: visual analysis tool for ranking and exploring genes, cell lines, and tissue samples. bioRxiv..

[25] Han S, Kim D, Kim Y (2018). CAS-viewer: web-based tool for splicing-guided integrative analysis of multi-omics cancer data. BMC Med Genomics.

[26] Gao J, Aksoy BA, Dogrusoz U (2013). Integrative analysis of complex cancer genomics and clinical profiles using the cBioPortal. Sci Signal..

[27] Cerami E, Gao J, Dogrusoz U (2012). The cBio cancer genomics portal: an open platform for exploring multidimensional cancer genomics data. Cancer Discov.

[28] Coarfa C, Pichot C, Jackson A (2014). Analysis of interactions between the epigenome and structural mutability of the genome using Genboree Workbench tools. BMC Bioinform.

[29] Schäfer M, Klein HU, Schwender H (2017). Integrative analysis of multiple genomic variables using a hierarchical Bayesian model. Bioinformatics.

[30] Rohart F, Gautier B, Singh A (2017). mixOmics: an R package for ‘omics feature selection and multiple data integration. PLoS Comput Biol.

[31] Silverbush D, Cristea S, Yanovich G (2018). ModulOmics: integrating multi-omics data to identify cancer driver modules. bioRxiv.

[32] Tuncbag N, Gosline SJ, Kedaigle A (2016). Network-based interpretation of diverse high-throughput datasets through the omics integrator software package. PLoS Comput Biol.

[33] Cline MS, Craft B, Swatloski T (2013). Exploring TCGA pan-cancer data at the UCSC cancer genomics browser. Sci Rep.

[34] Kannan L, Ramos M, Re A (2016). Public data and open source tools for multi-assay genomic investigation of disease. Brief Bioinform.

[35] Silva TC, Colaprico A, Olsen C (2016). TCGA workflow: analyze cancer genomics and epigenomics data using Bioconductor packages. F1000Res.

[36] Bodalal Z, Trebeschi S, Nguyen-Kim TDL, Schats W, Beets-Tan R (2019). Radiogenomics: bridging imaging and genomics. Abdom Radiol.

[37] Haas R, Zelezniak A, Iacovacci J (2017). Designing and interpreting ‘multi-omic’ experiments that may change our understanding of biology. Curr Opin Struct Biol.

[38] Chakraborty S, Hosen MI, Ahmed M (2018). Onco-multi-OMICS approach: a new frontier in cancer research. Biomed Res Int.

[39] Mirza B, Wang W, Wang J (2019). Machine learning and integrative analysis of biomedical big data. Genes.

[40] Gevaert O, Xu J, Hoang CD (2012). Non–small cell lung cancer: identifying prognostic imaging biomarkers by leveraging public gene expression microarray data—methods and preliminary results. Radiology.

[41] Mazurowski MA, Zhang J, Grimm LJ, Yoon SC, Silber JI (2014). Radiogenomic analysis of breast cancer: luminal B molecular subtype is associated with enhancement dynamics at MR imaging. Radiology.

[42] Guo W, Li H, Zhu Y (2015). Prediction of clinical phenotypes in invasive breast carcinomas from the integration of radiomics and genomics data. J Med Imaging.

[43] Karlo CA, Di Paolo PL, Chaim J (2014). Radiogenomics of clear cell renal cell carcinoma: associations between CT imaging features and mutations. Radiology.

[44] Shofty B, Artzi M, Ben Bashat D, Liberman G, Haim O, Kashanian A, Bokstein F, Blumenthal DT, Ram Z, Shahar T (2018). MRI radiomics analysis of molecular alterations in low-grade gliomas. Int J Comput Assist Radiol Surg.

[45] Li Y, Liu X, Xu K, Qian Z, Wang K, Fan X, Li S, Wang Y, Jiang T (2008). MRI features can predict EGFR expression in lower grade gliomas: a voxel-based radiomic analysis. Eur Radiol.

[46] Mazurowski MA (2015). Radiogenomics: what it is and why it is important. J Am Coll Radiol.

[47] Meng C, Zeleznik OA, Thallinger GG (2016). Dimension reduction techniques for the integrative analysis of multi-omics data. Brief Bioinform.

[48] Edwards NJ, Oberti M, Thangudu RR (2015). The CPTAC data portal: a resource for cancer proteomics research. J Proteome Res.

[49] Wilson S, Fitzsimons M, Ferguson M (2017). Developing cancer informatics applications and tools using the NCI genomic data commons API. Cancer Res.

[50] Zhang J, Baran J, Cros A (2011). International Cancer Genome Consortium Data Portal—a one-stop shop for cancer genomics data. Database..

[51] Clark K, Vendt B, Smith K (2013). The Cancer Imaging Archive (TCIA): maintaining and operating a public information repository. J Digit Imaging.

[52] Zheng-Bradley X, Flicek P (2017). Applications of the 1000 Genomes Project resources. Brief Funct Genomics..

[53] Katako A, Shelton P, Goertzen AL (2018). Machine learning identified an Alzheimer’s disease-related FDG-PET pattern which is also expressed in Lewy body dementia and Parkinson’s disease dementia. Sci Rep.

[54] Pont-Sunyer C, Tolosa E, Caspell-Garcia C (2017). The prodromal phase of leucine-rich repeat kinase 2-associated Parkinson disease: clinical and imaging Studies. Mov Disord.

[55] Kang UJ, Goldman JG, Alcalay RN (2016). The BioFIND study: characteristics of a clinically typical Parkinson’s disease biomarker cohort. Mov Disord.

[56] Payakachat N, Tilford JM, Ungar WJ (2016). National Database for Autism Research (NDAR): big data opportunities for health services research and health technology assessment. Pharmacoeconomics.

[57] Gwinn K, David KK, Swanson-Fischer C (2017). Parkinson’s disease biomarkers: perspective from the NINDS Parkinson’s Disease Biomarkers Program. Biomark Med.

[58] Hodes RJ, Buckholtz N (2016). Accelerating Medicines Partnership: Alzheimer’s Disease (AMP-AD) knowledge portal aids Alzheimer’s drug discovery through open data sharing. Expert Opin Ther Targets.

[59] Butkiewicz M, Blue EE, Leung YY (2018). Functional annotation of genomic variants in studies of late-onset Alzheimer’s disease. Bioinformatics.

[60] Fonseca CG, Backhaus M, Bluemke DA (2011). The Cardiac Atlas Project—an imaging database for computational modeling and statistical atlases of the heart. Bioinformatics.

[61] Giffen CA, Carroll LE, Adams JT (2015). Providing Contemporary Access to Historical Biospecimen Collections: development of the NHLBI Biologic Specimen and Data Repository Information Coordinating Center (BioLINCC). Biopreserv Biobank.

[62] Liu B, Madduri RK, Sotomayor B (2014). Cloud-based bioinformatics workflow platform for large-scale next-generation sequencing analyses. J Biomed Inform.

[63] Ye Z, Kalloo FS, Dalenberg AK (2013). An electronic medical record-linked biorepository to identify novel biomarkers for atherosclerotic cardiovascular disease. Glob Cardiol Sci Pract.

[64] Craig T, Smelick C, Tacutu R (2015). The Digital Ageing Atlas: integrating the diversity of age-related changes into a unified resource. Nucleic Acids Res.

[65] Tryka KA, Hao L, Sturcke A (2014). NCBI’s database of genotypes and phenotypes: dbGaP. Nucleic Acids Res.

[66] Lappalainen I, Almeida-King J, Kumanduri V (2015). The European Genome-phenome Archive of human data consented for biomedical research. Nat Genet.

[67] Barrett T, Wilhite SE, Ledoux P (2012). NCBI GEO: archive for functional genomics data sets—update. Nucleic Acids Res.

[68] Tacutu R, Thornton D, Johnson E (2018). Human Ageing Genomic Resources: new and updated databases. Nucleic Acids Res.

[69] Kodama Y, Mashima J, Kosuge T (2018). DNA Data Bank of Japan: 30th anniversary. Nucleic Acids Res.

[70] Bakas S, Akbari H, Sotiras A, Bilello M (2017). Segmentation labels and radiomic features for the pre-operative scans of the TCGA-LGG collection. Cancer Imaging Archiv..

[71] Morris E, Burnside E, Whitman G (2014). Using computer-extracted image phenotypes from tumors on breast mri to predict stage. Cancer Imaging Archiv..

[72] Bakas S, Akbari H, Sotiras A, Bilello M (2017). Segmentation labels and radiomic features for the pre-operative Scans of the TCGA-GBM collection. Cancer Imaging Archiv..

[73] Singh A, Gautier B, Shannon CP (2018). DIABLO: from multi-omics assays to biomarker discovery, an integrative approach. bioRxiv.

[74] Tenenhaus A, Philippe C, Guillemot V (2014). Variable selection for generalized canonical correlation analysis. Biostatistics.

[75] Argelaguet R, Velten B, Arnol D (2018). Multi-omics factor analysis—a framework for unsupervised integration of multi-omics data sets. Mol Syst Biol.

[76] Lock EF, Hoadley KA, Marron JS (2013). Joint and individual variation explained (JIVE) for integrated analysis of multiple data types. Ann Appl Stat.

[77] Ramos M. curatedTCGA Data: Curated Data From The Cancer Genome Atlas (TCGA) as MultiAssayExperiment Objects. R package version 1.3.5.

[78] GDC Sample Type Codes. https://gdc.cancer.gov/resources-tcga-users/tcga-code-tables/sample-type-codes. Accessed 5 July 2019.

[79] Morgan M, Obenchain V, Hester J et al. SummarizedExperiment: SummarizedExperiment container. R package version 1.10.1.

[80] Bioconductor page of MultiAssayExperiment. http://bioconductor.org/packages/release/bioc/html/MultiAssayExperiment.html. Accessed 5 July 2019.

[81] Lex A, Gehlenborg N, Strobelt H (2014). UpSet: visualization of Intersecting Sets. IEEE Trans Visual Comput Graphics.

